# Inundation2Depth: A multi-source dataset for floodwater depth estimation in urban areas

**DOI:** 10.1016/j.dib.2025.112347

**Published:** 2025-12-01

**Authors:** Jeffrey Blay, Yared Gebregziabher, Manoj K Jha, Leila Hashemi Beni

**Affiliations:** a Department of Built Environment, North Carolina A&T State University, 1601 E Market St., Greensboro, NC 27401, USA; b Department of Computational Data Science and Engineering, North Carolina A&T State University, 1601 E Market St., Greensboro, NC 27401, USA; c Department of Civil, Architectural and Environmental Engineering, North Carolina A&T State University, 1601 E Market St., Greensboro, NC 27401, USA

**Keywords:** Remote sensing, Deep learning, Natural disasters, Risk modelling, Hydrodynamic modelling, Machine learning, Geospatial engineering

## Abstract

Floods impose severe risks in urban areas, yet operational mapping often stops at inundation extent rather than depth, which is critical for assessing and managing accessibility, and risk. Progress in deep learning offers a path forward but is constrained by the scarcity of large quantity, georeferenced depth datasets that are well labelled. Inundation2Depth dataset pairs inundation extent-depth labels derived from aerial imagery and LiDAR (Light Detection and Ranging)-based DTMs (Digital Terrain Models) under hydrostatic assumptions (water-surface elevation relative to terrain). The dataset encompass 12 flood affected areas across North and South Carolina in the Southeastern United States, covering 24,649.88 acres with diverse environmental characteristics. Data acquisition spans the period between 2016 and 2018, corresponding to two major hurricane flood events that significantly impacted the Carolinas. The sources include complementary layers from multi-sensor remote sensing imagery (thus, Optical and LiDAR point cloud data), which were preprocessed through standard correction, normalization, and georeferencing steps to ensure spatial consistency across scenes. Data are provided as scene-level raster and 256×256 tiles, available in both raw and normalized versions with consistent naming to support direct integration in machine/deep learning pipelines. It constitutes a total of 5925 overlapping tiles. This dataset’s unique characteristics, such as spatial diversity and standardized format, make it valuable for developing and evaluating flood detection, segmentation, and damage assessment models. *Inundation2Depth* lowers the data barrier for GeoAI research on urban flood severity and promotes comparability across methods and study areas. The generated dataset was validated through a hydrodynamic modeling approach using HEC-RAS Rain-on-Grid tool.

Specifications Table

**Table:** 

Subject	Earth & Environmental Sciences/ GeoAI
Specific subject area	Geospatial data engineering techniques for post-event flood depth modelling/segmentation
Type of data	.Geotiff (image files)
Data collection	Post-event aerial imagery is acquired at 2500 – 5000 ft (762–1524 m) with Trimble Digital Sensor System (35–50 cm GSD) (NOAA Storms Archive). Airborne LiDAR are collected near 10,000 ft (3048 m) and processed to DTM (NC Emergency Management; USGS 3DEP). High-water marks are surveyed in the field by trained crews’ post-flood (USGS (United States Geological Survey)). Sites are selected for cloud-free imagery, co-registered LiDAR, and verified HWMs. Depth labels are derived hydrostatically (water surface–DTM). All generated layers are transformed (where appropriate) and min–max scaled to 0–1 for unit consistency. Processing involved the use of ArcGIS/ArcPy, GDAL, and Python. Input data for HEC-RAS hydrodynamic analysis includes: Terrain: 3 ft (1 m) resolution Digital Elevation Model (DEM) from North Carolina, NC—OneMap, further resampled to 1.5 ft (0.5 m) resolution (NC OneMap). Rainfall: Gridded daily precipitation data from NOAA’s Stage IV product (NOAA’s Stage IV product). Land use land cover database was collected from USGS National Land Cover Database (USGS NLC Database). Soils data set was acquired from USDA-NRCS (United States Department of Agriculture – Natural Resources Conservation Service) (USDA-NRCS soils data).
Data source location	Country: USA Region: The Carolinas (North and South), Southeast USA Cities: Lumberton, Princeville, Goldsboro, Kinston, Hancheys Store, Greenville, Chinquaqin, Wallace, Nichols
Data accessibility	Repository name: Inundation2Depth Dataset Data identification number: 10.5281/zenodo.17308287 Direct URL to data: https://zenodo.org/records/17,308,287?preview=1&token=eyJhbGciOiJIUzUxMiJ9.eyJpZCI6IjJiNjcwYzYzLTkxNzAtNDg3OC1hYTZkLWExOWMwNTRjMzlhZCIsImRhdGEiOnt9LCJyYW5kb20iOiI5YmYwNjdlMjI0ZGI3YTRhMWM5NGM3MTZlYTE3MTMxMiJ9.4Yb0b9fRvJ6OkEsAAgjWzdAQXJ_FdO8dKulAt3pke3p8I3CIlJSJ1fhlwm3Y4a06nsGiyBH25sGSpWywLKPy-A
Related research article	NONE

## 1 Value of the Data

• This dataset constitutes curated urban flood scenes, that pair inundation extent with georeferenced depth labels derived under hydrostatic assumptions (water-surface elevation relative to terrain), providing 3D flood maps, for analysis and risk assessment.
• The dataset is distributed in two synchronized versions: Raw (original units) and Normalized (log/cube-root, where appropriate, and scaled to 0–1), which let researchers reproduce preprocessing, run ablations, and use data for diverse workflows.
• The dataset is provided as scene-level raster and 256×256 tile products with simple, consistent filenames for seamless ML/DL training, validation, and patch-based experiments.
• The dataset standardizes inputs, labels, and metadata to enable reproducible benchmarking and cross-method comparisons in urban flood-depth inference.
• The dataset is suitable for training depth-regression models; conditioning/validation of hydrostatic simulations; transfer learning; feature engineering studies; and education/reproducible tutorials on geospatial Machine/Deep Learning.

## 2 Background

Floods remain a leading urban hazard. For response and damage assessment, rapidly identifying inundated areas and mapping how deep the inundated water is, not just where it is, provides actionable intelligence on flood risk to people and assets [1,2]. Remote sensing enables timely, wide-area flood mapping, and recent improvements in deep learning has advanced flood inference in complex built environments [3].

Substantial progress has been made in inundation extent mapping from remote sensing sources, often through machine/deep learning and image segmentation. Yet, estimating floodwater depth is constrained, largely due to the scarcity of well-annotated, georeferenced datasets that pair ground-referenced depth information with inundation extent for training and evaluation [4]. This gap limits operational severity mapping and risk modeling, especially in data-scarce regions and urban areas where rapid flood management and recovery is critical.

Inundation2Depth addresses this need by curating post-event urban scenes where inundated water can be approximated as at rest, enabling depth labels, derived under hydrostatic principles (water-surface elevation relative to terrain). The dataset couples post-flood imagery with LiDAR-based DTM and terrain derivatives, harmonized flood-extent masks, and hydrostatic depth annotations, with documented QA/QC. By standardizing inputs, labels, and metadata, it provides a foundation for developing and benchmarking deep learning models that infer floodwater depth, not just extent, to deliver more reliable flood-severity mapping and decision-ready tools during real events.

## 3 Data Description

The dataset consists of multi-sourced geospatial raster layers capturing post‐flood conditions, terrain attributes, and landcover/landuse characteristics for various flood affected areas (see section 4). All layers are co-registered, share site–based filenames, and are provided as GeoTIFFs in folders of the linked repository (“{*sitename}.tiff”)*. A top-level ReadMe.txt lists layer-specific metadata (CRS, units, value ranges, and NoData).

The datasets are provided in two main versions:

• **Raw Data:** Pre-processed source raster layers for all features in their original units.
• **Normalized Data:** Log or cube-root transforms as appropriate, followed by per-layer min–max scaling to a common range ([0–1]).

Each data version contains scene and tile products:

• **Full Scenes:** Wall-to-wall raster layers for each feature and sample site (see layer subfolders).
• **Tiles:** 256×256-pixel patches (∼1 m resolution) generated from the full scenes for model training/evaluation. Tiles are uniformly named as (“*{sitename}_tile_{tileID}.tiff* ”).

**Fig. 1:**
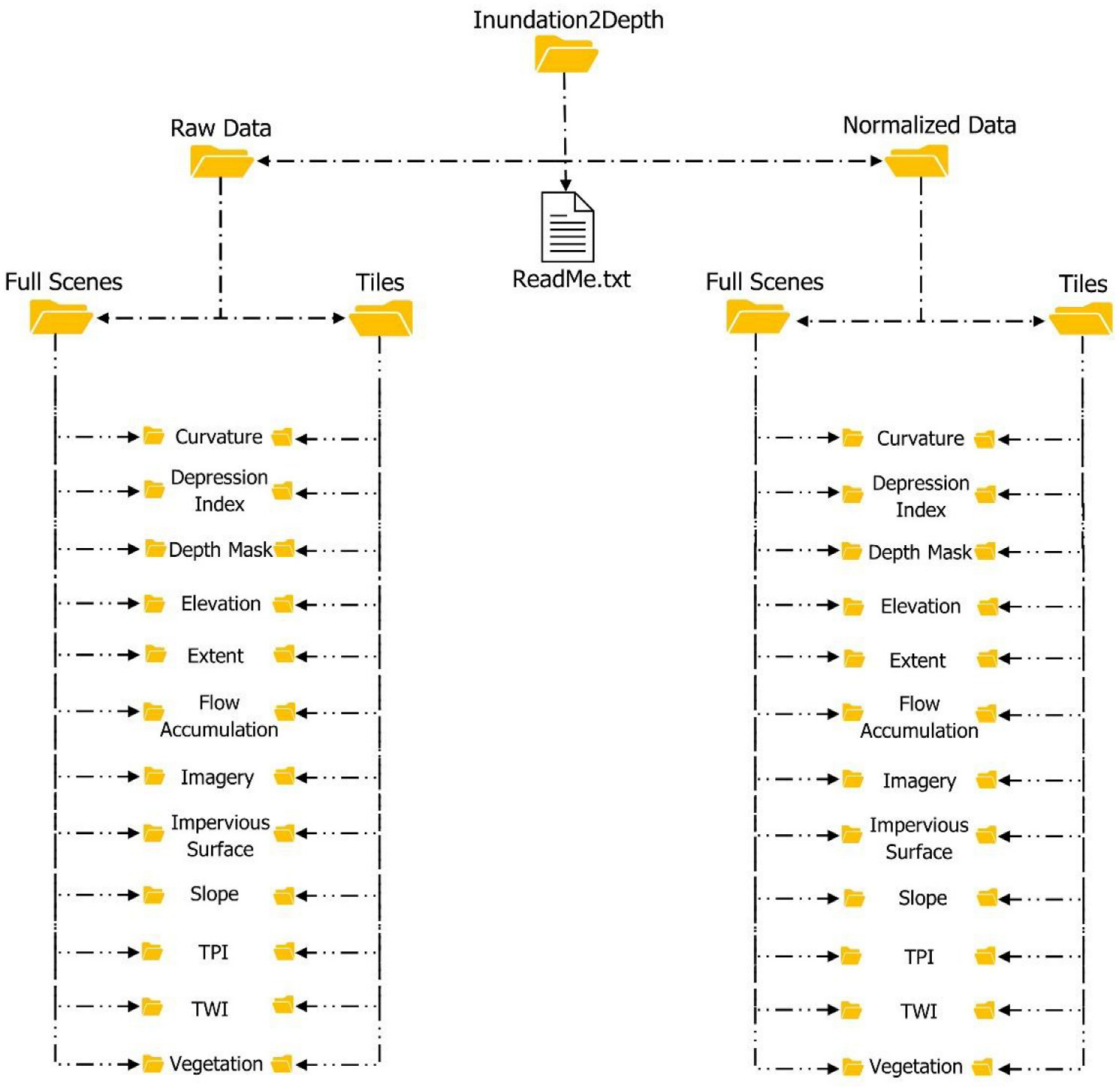
Organization of Inundation2Depth repository.

To clarify how files are organized for different workflows, Fig. 1 illustrates the Inundation2Depth repository layout. Parallel Raw and Normalized branches with identical layer subfolders, each provided as Full Scenes and 256×256 Tiles. It is organized to ensure easy navigation from scenes to training ready tiles for each data version. Table 1 provides information on the layer subfolders, their purpose, and units.

**Table 1:** Content of layer subfolders in the Inundation2Depth data repositories.

Layer (subfolder)	Description	Units / Encoding
Imagery	Post-event aerial imagery (multi-band), co-registered with extent/DTM. Channel order is RGB.	DN 0–255
Elevation (DTM)	Ground elevation surface.	ft
Depth Mask	Ground-truth flood depth raster aligned to inundated extent.	ft (float32); dry=0
Extent	Inundation mask used for conditioning	Binary: 1 = flood, 0 = no flood
Slope	Terrain slope, computed from DTM.	degrees (deg)
Curvature (plan)	Planform curvature; identifies convergent/divergent flow. negative (concave) values set to 0 to emphasize convex (divergent) slopes.	1/meters (m⁻¹)
Depression Index	Indicator of local sinks/ponding potential.	unitless
Flow Accumulation	Upslope contributing area. Formular= D8/D∞	unitless
TPI (Topographic Position Index)	Elevation relative to neighborhood (ridges/valleys). Window size = 11×11. negative (valley-like) values set to 0 to emphasize ridge-like positions.	unitless
TWI (Topographic Wetness Index)	Proxy for potential water accumulation at a location. Formular= ln(a / tanβ). *a*= area, tanβ=slope gradient.	unitless
Impervious Surface	Imperviousness mask (roads, buildings, concrete).	Binary: 1 = impervious, 0 = pervious
Vegetation	Vegetation mask (trees/grass/herbaceous).	Binary: 1 = vegetation, 0 = non-vegetation

## 4 Experimental Design, Materials and Methods

### 4.1 Data collection sites

**Fig. 2:**
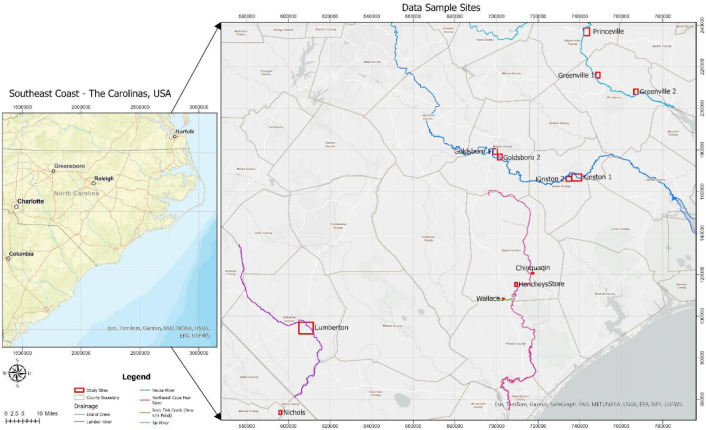
Map of sampling sites.

Inundation2Depth constitutes a set of flood impacted built environments in USA (North Carolina and South Carolina), affected by two hurricane disasters: Hurricanes Matthew (2016) and Florence (2018). The sample sites include 12 urban and peri‑urban areas totaling 24,649.88 acres of land coverage, with elevations spanning 5.49–45.11 m (median 13.41 m). The goal was to capture diverse urban forms and drainage settings to ensure the datasets includes a broad range of flood behaviors. This variability supports training and evaluation of depth-estimation approaches intended to generalize across different urban forms and drainage contexts. These areas are located within the flood plains of various natural drainage systems within the states as shown in Fig. 2 and described in Table 2. The flood prone sites were chosen based on:

1. The availability of co-registered/georeferenced post-event aerial imagery and LiDAR-derived ground elevation (DTM).
2. Visual documentation of substantial inundation with built areas.
3. Availability of field indicators (e.g., high-water marks) suitable for hydrostatic depth derivation calibration.

**Table 2:** Data collection site catalogue (grouped by event).

Site	Area (acres)	Elevation (m)	Nearest Drainage
**Hurricane Matthew - 23,836.52 acres total**			
Lumberton	9496.55	45.11	Lumber River
Kinston 1	3886.70	19.20	Neuse River
Kinston 2	1465.11	13.72	Neuse River
Goldsboro 1	1510.81	28.65	Neuse River
Goldsboro 2	1536.73	23.77	Neuse River
Greenville 1	1377.24	10.97	Tar River
Greenville 2	1347.99	9.75	Tar River
Princeville	2597.82	16.15	Tar River
Nichols	617.57	5.49	Lumber River
**Hurricane Florence - 813.36 acres total**			
Hancheys Store	534.85	12.80	Island Creek
Chinquapin	217.41	13.11	Northeast Cape Fear River
Wallace	61.10	11.58	Little Rockfish Creek

The following sections describe the data sources, data generation pipeline, technical validation procedures, and guidance for data use.

### 4.2 Data sources

Four complementary datasets were compiled: post-flood aerial imagery, LiDAR-derived DTM, field-surveyed high-water marks, and flood depth maps from hydrodynamic models (Table 3).

**Table 3:** Summary datasets used in this study and their attributes.

Dataset	Source	Dates	Resolution / Type	Coverage	Purpose
Post-flood aerial imagery	NOAA Storms Archive	Matthew: Oct 10–15, 2016; Florence: Sep 18, 2018	∼ 50 cm RGB orthophotos	Affected settlement areas in this study	Post-event surface conditions
Elevation (LiDAR → DTM)	NC Emergency Management; USGS 3DEP	Pre-event (2014)	∼1 m DEM + derivatives	Study sites (NC/SC)	Terrain and features (slope, curvature, TWI, etc.)
High-Water Marks (HWMs)	USGS (United States Geological Survey)	Post-storm surveys	2 Points per 1 sq. meter observations	Near study areas	Reference for depth mask evaluation
HEC-RAS depth maps	Internally generated	Oct 1–31, 2016	∼ 1 m	Selected validation sites	Reference for depth mask evaluation

### 4.3 Data generation

The project workflow consists of a series of steps to process and integrate the various geospatial datasets to derive flood extent and depth products, to validate and prepare uniform tiles for modelling.

#### 4.3.1 Step 1: Flood extent delineation

**Fig. 3:**
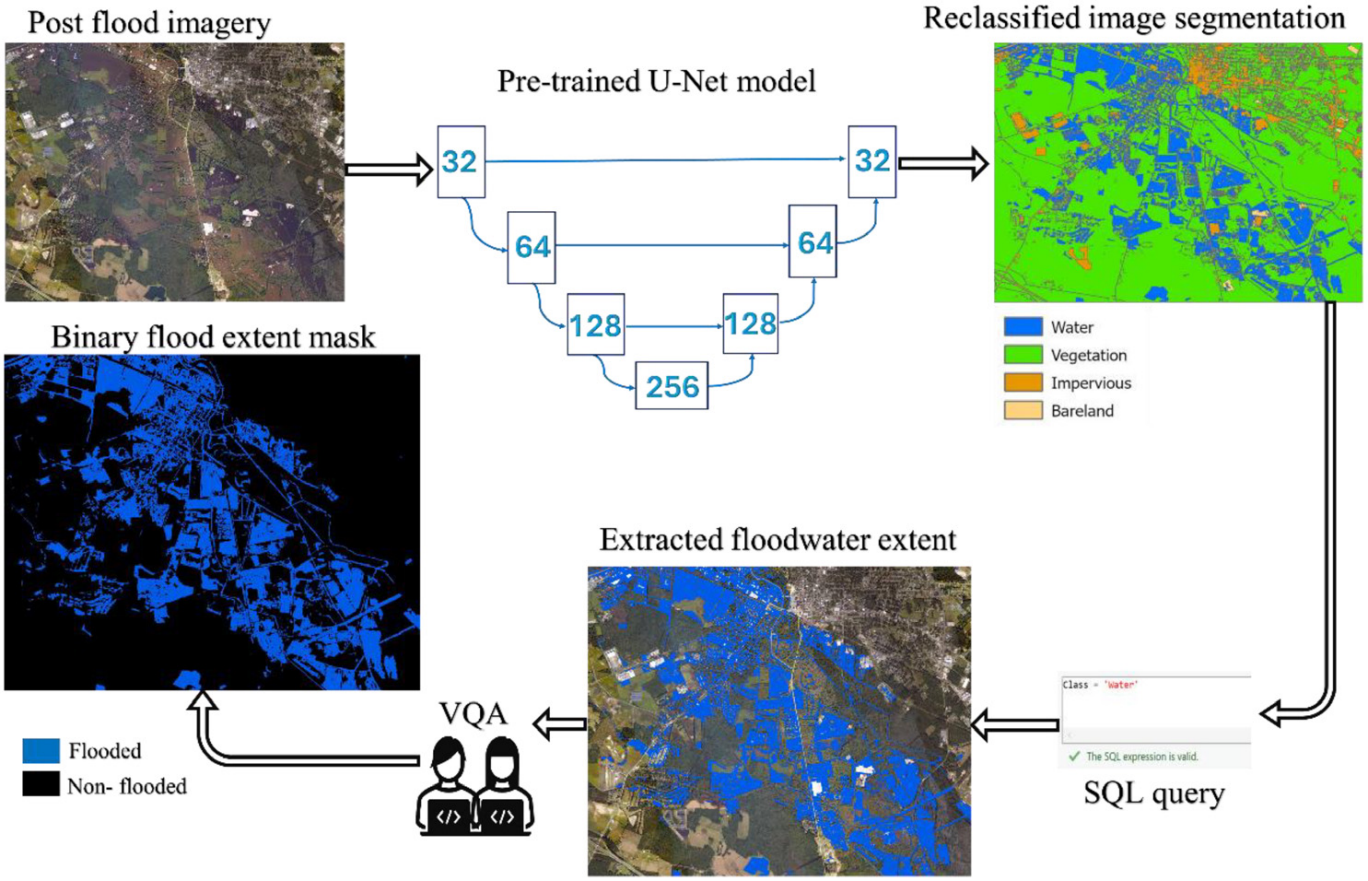
Flood extent delineation workflow.

Post-event aerial imagery was segmented with a pretrained U-Net from ESRI to identify “flooded” pixels. The model originally outputs 9 classes, which were reclassified into 4 main classes, including a water class. The flooded class was extracted via SQL query and converted to a binary raster (1=flooded, 0=non-flooded), at a spatial resolution of 1 m (∼ 3 ft). Then, a standard cartographic cleanup (e.g. hole filling, small-patch removal) and topology checks were performed, and the mask was validated by visual inspection against high resolution basemaps in QGIS and ArcGIS Pro to correct commission and omission errors. The final extent mask is then aligned to the project grid (*NAD 1983 StatePlane North Carolina FIPS 3200 (US Feet)*) for downstream use (Fig. 3).

#### 4.3.2 Step 2: Terrain feature generation from lidar-derived DTM

**Fig. 4:**
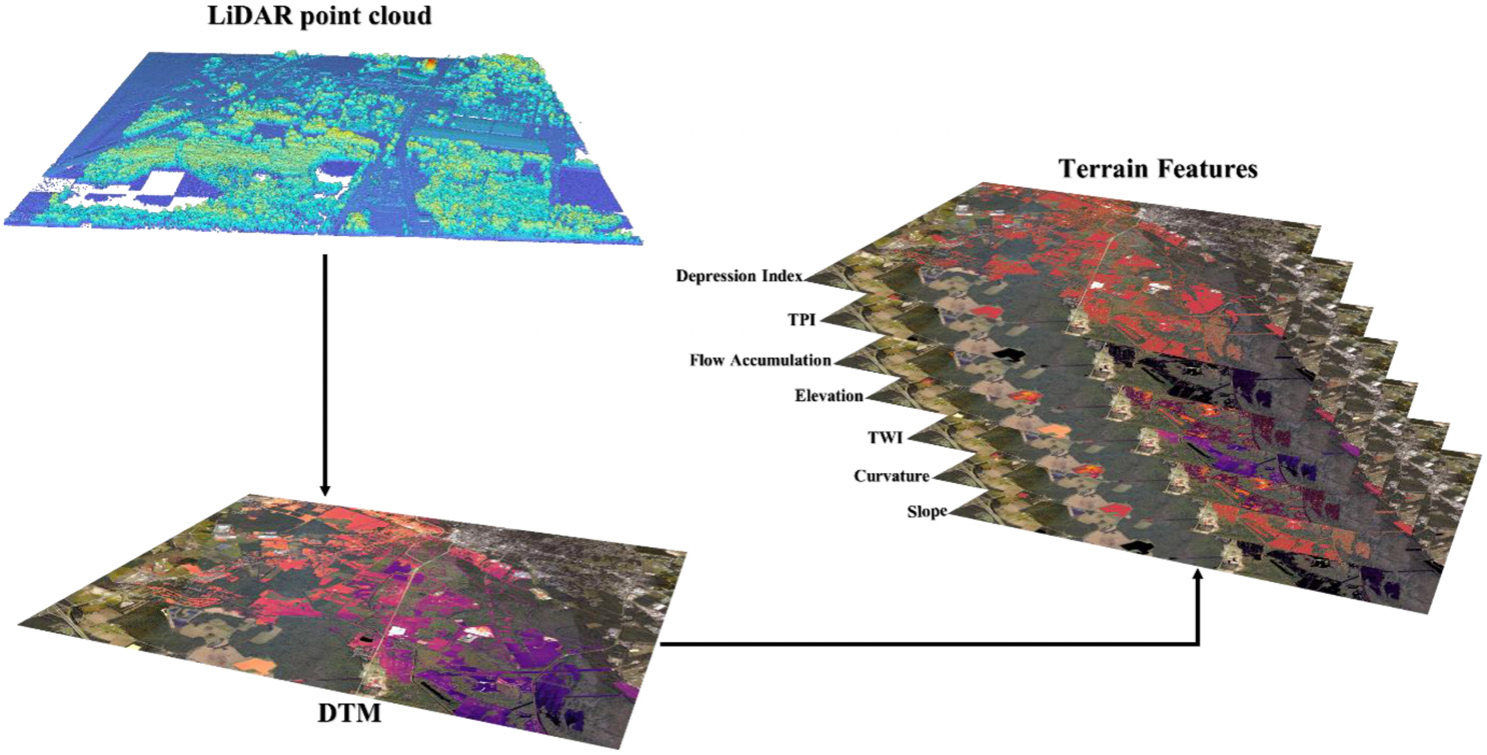
Workflow for generating terrain features.

A custom python library (“*ezprocess*”) was employed to process LiDAR 3D point cloud to generate DTM for each study area. The derived DTM was then co-registered to the stipulated grid (“*NAD 1983 StatePlane North Carolina FIPS 3200 (US Feet)”*) and was subsequently used to compute elevation and secondary terrain variables: slope, curvature(plan), Topographic Wetness Index (TWI), topographic position index (TPI), flow accumulation, and depression index. Each layer was clipped by the extent mask to restrict values to inundated areas, with non-flood pixels encoded as 0 for consistency. All layers were validated to remove and smoothen erroneous, as well as peak pixel noise values. Finally, each layer was standardized to a common CRS (Coordinate Reference System), pixel size and NoData policy (Fig. 4).

#### 4.3.3 Step 3: Flood depth mask generation

The floodwater depth mask was generated under hydrostatic conditions as captured by the post-event imagery by pairing the validated flood-extent raster with the co-registered DTM aligned to common coordinate reference system and resolution. Within each connected inundation region, a piecewise-smooth water-surface elevation field *η(x,y)* consistent with water at rest and independent of sub-grid roughness, was computed. Flood depth at each pixel was derived as

H(x,y)=max(0,η(x,y)−z(x,y))

where z(x,y) is ground elevation.

**Fig. 5:**
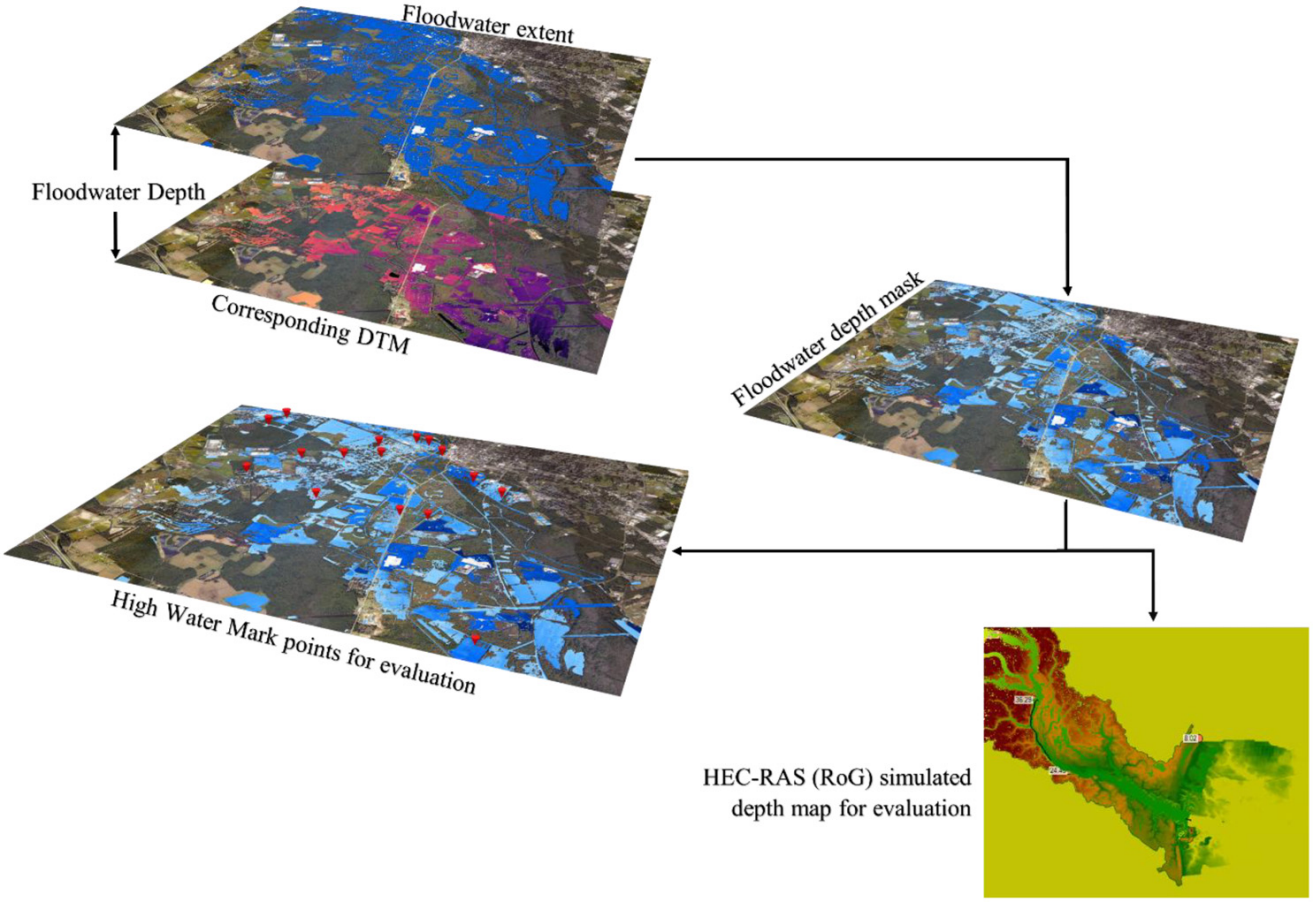
Workflow for generating and evaluating flood depth mask.

To reduce speckle and isolated peaks while preserving boundary line transitions, a light, edge-preserving smoothing was applied using a 3 × 3 gaussian smoothing kernel to the depth field. Implausible values were removed using thresholds informed by available ground truth, i.e. high-water marks and HECRAS-based depth maps, and then water depth maps were clipped to site-appropriate ranges to maintain physical interpretability. Pixels outside the mapped extent were encoded as zero for the water depth. The resulting raster thus reflects a hydrostatically consistent, non-negative estimate of post-floodwater depth suitable for downstream modelling and evaluation (Fig. 5).

#### 4.3.4 Step 4: Feature transformations and normalization

There are two versions of the datasets, a raw stack, which allows independence for users to perform their own processing and a normalized stack, designed to streamline modelling and analysis. With the normalized stack, targeted transformations were applied to stabilize heavily-skewed features and improve comparability prior to scaling: curvature received a cube-root transform (following prior practice e.g [5]), while depression index and flow accumulation were log-transformed. After these feature-specific transforms, all continuous predictors were then min–max normalized to [0,^1^] on a per-feature basis using dataset-wide minima and maxima, and binary layers (e.g., extent masks) were left unchanged (Table 4). The data-wide minimum and maximum are defined as the smallest and largest values, respectively, computed across the entire dataset in all sites. This approach provides a uniform 0–1 scale for normalization across sites, and avoiding per-site rescaling that can distort magnitudes. The normalized stack is provided as a separate companion to the raw stack, with stored scaling parameters to enable exact inversion or reuse.

**Table 4:** Transform and normalization attributes of feature layers.

Layer	Transformation	Data-wide Minimum	Data-wide Maximum	Normalization
Elevation	None	0.0	147.67	Min–max to [0,1]
Depth	None	0.0	28.68	Min–max to [0,1]
Curvature (plan)	Clamp negatives → cube-root	0.0	6.25	Min–max to [0,1]
TPI	Clamp negatives	0.0	1.0	Min–max to [0,1]
TWI	None	0.0	30.08	Min–max to [0,1]
Depression Index	log1p	0.0	2.88	Min–max to [0,1]
Flow Accumulation	log1p	0.0	10.84	Min–max to [0,1]
Slope	log1p	0.0	4.38	Min–max to [0,1]
Imagery	None	0.0	255	Min–max to [0,1]

All minimum and maximum values are computed after the stated transform and used for global min–max scaling to [0,1]. “log1p” denotes log(1 + *x*) (natural log). 0= dry areas/ no depth.

#### 4.3.5 Step 5: Tile creation

**Fig. 6:**
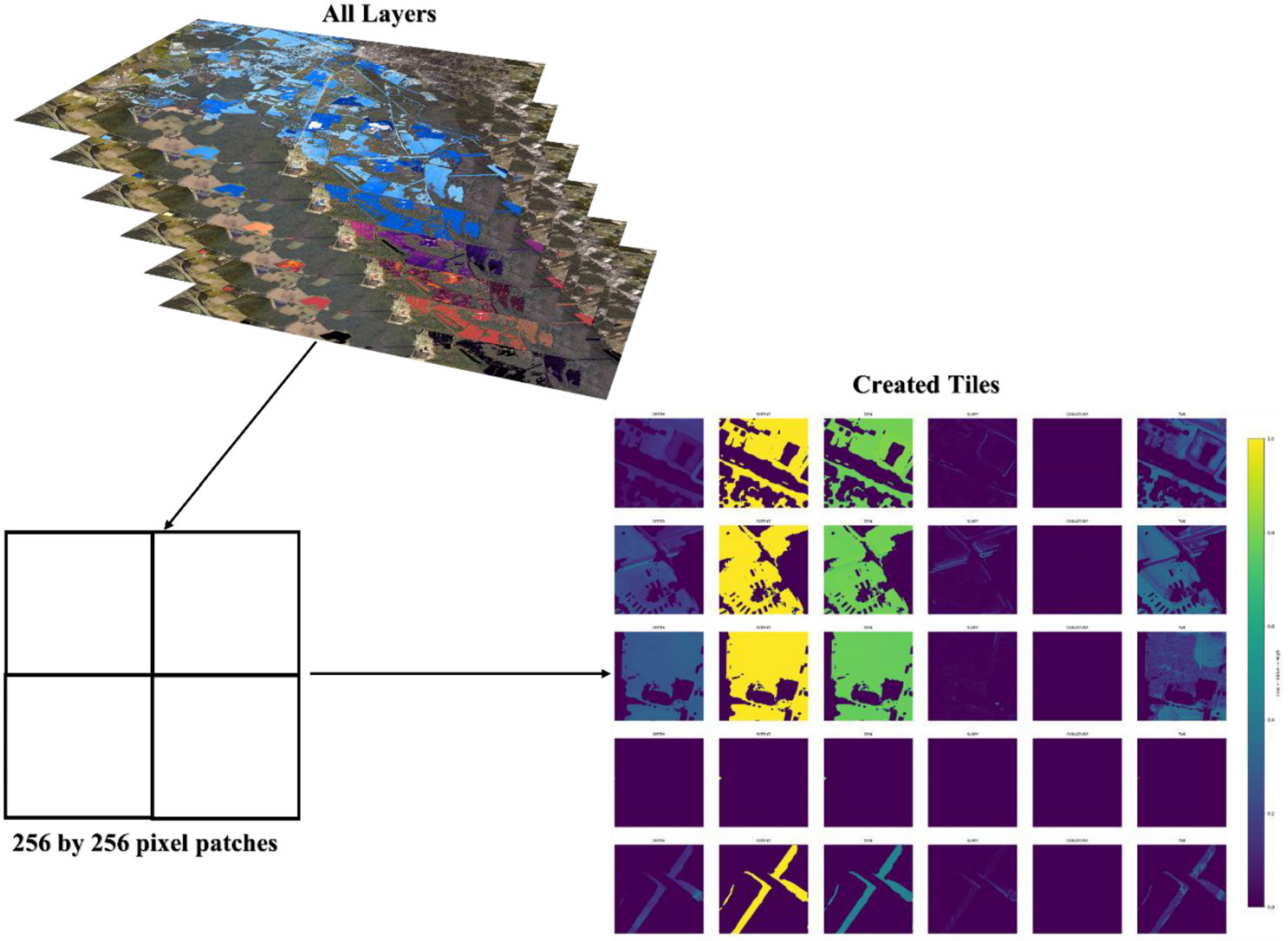
Tile creation workflow.

All co-registered layers were tiled using a custom python library called ezprocess v0.1.0, extracting fixed windows of 256×256 pixels with 50 % overlap (stride =128px). The identical window grid was applied to every raster (features, imagery, extent, and depth), preserving strict pixel-wise alignment within each stack. Both the raw and the normalized versions were tiled identically with tiles named by site and row/column indices to enable deterministic geolocation and reassembly (Fig. 6). In total, 5925 overlapping tiles were generated.

### 4.4 Technical validation

This study performed validation using both quantitative and qualitative approaches.

**Quantitative validation.** Site-level and aggregate metrics for floodwater depth, including RMSE, MAE, bias, R-square, were computed on matched spatial supports and summarized in tables for selected sites with available ground reference data. Water depth predictions were benchmarked against two independent references: field-surveyed High-Water Marks and HEC-RAS Rain-on-Grid (RoG) maximum-depth rasters, after aligning references to the analysis grid (nearest-pixel sampling for HWMs; resampling and co-registration for RoG). For quantitative validation, physics-based water depth fields were generated with HEC-RAS 6.5 Rain-on-Grid 2D hydrodynamic modeling for the Tar River Basin case study covering Towns of Princeville and Greenville from 1 to 31 October 2016. Model Inputs included a 0.5 m (1.5 ft) DTM from NC OneMap, gridded daily precipitation data from NOAA’s Stage IV product, NLCD land cover data (27 classes) to parameterize Manning’s *n*, and the USDA-NRCS soils data to delineate hydrologic soil groups for infiltration modeling and to assign Curve Numbers (CN) and initial abstraction ratios. The computational mesh used a 152 m (500 ft) base grid refined to 30.5 m (∼100 ft) along channels and urban corridors. Model calibration leveraged eight USGS gaging stations (peak discharge and depth values) and post-event high-water marks; the calibrated model reproduced observations with strong metrics in agreement with ground observations, achieving R² = 0.87 and NSE = 0.84 for maximum depth, and R² = 0.92 and NSE = 0.80 for peak discharge.

**Fig. 7:**
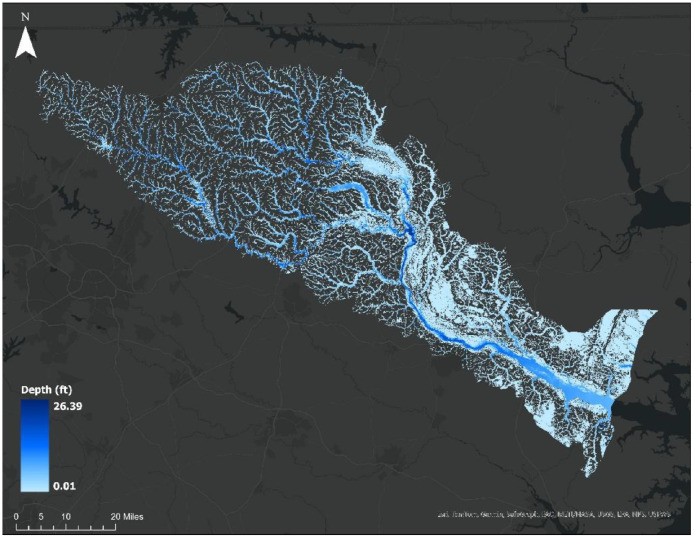
RoG maximum depth map of Hurricane Mathew (2016) on Tar-Pamlico river basin.

RoG maximum-depth rasters were exported, clipped to sample site boundaries, and aligned to the analysis grid, yielding spatially continuous, event-consistent depth fields that serve as reference datasets for benchmarking image-derived water depth prediction. These references complement point HWMs by providing basin-wide continuity and are summarized alongside site-level metrics, including the overall performance of the pretrained segmentation model (Fig. 7; Table 5–6). All metrics were computed in native depth units with consistent no-data masking and shared valid-pixel intersection. Finally, a geospatial uniformity a was verified across all layers (identical CRS, pixel size, spatial extent).

**Table 5:** Evaluation metrics of flood depth mask against reference datasets for selected validation sites in each basin.

Validation Site	RMSE (m)	MAE (m)	River
Lumberton	0.76	0.50	Lumber river
Goldsboro 1	0.09	0.07	Neuse river
Princeville	0.38	0.34	Tar river
Chinquaqin	0.77	0.59	Northeast cape fear river
Hancheys Store	0.56	0.46	Island creek
Wallace	0.72	0.71	Little rockfish creek

**Table 6:** Per class segmentation metrics of the pretrained U-Net model (*source:**ESRI*).

Class	Precision	Recall	F1 Score
Water	0.94	0.93	0.93
Wetlands	0.82	0.76	0.79
Tree Canopy	0.90	0.93	0.92
Shrubland	0.52	0.19	0.27
Low Vegetation	0.86	0.87	0.86
Barren	0.67	0.51	0.58
Structures	0.81	0.85	0.83
Impervious Surfaces	0.74	0.69	0.71
Impervious Roads	0.76	0.81	0.79

**Qualitative validation.** Also, a structured visual inspections of depth and extent overlays were conducted on post-event imagery and terrain derivatives to confirm hydrostatic plausibility, boundary fidelity, and spatial coherence. Reviews focused on shoreline continuity, pooling in local depressions, consistency along transects, and alignment with mapped infrastructure and drainage. Also, all layer maps were inspected to identify systematic artifacts such as striping, stair-stepping, or over-smoothing. Non-flood areas were verified to be zero-coded throughout the layers (except for the imagery).

### 4.5 Data use

To streamline end-to-end experimentation, a lightweight Python library, *ezprocess*, is developed to operationalize this pipeline, from preprocessing, data exploration and feature importance, tiling and loading pipelines for training and evaluation. The library exposes a parameterized API for reproducible preprocessing, dataset manifests, train/val/test splits, and ready-to-use loaders for deep learning frameworks, with built-in QA checks (CRS/pixel size agreement, no-data handling). It can be installed *via pip install ezprocess* and is intended to support both training and independent evaluation with minimal overhead.

**Interoperability note:** Any externally derived flood-extent product (e.g., SAR-based delineations) can be substituted and paired with the Depth Mask provided for training or evaluating depth-estimation models.

### 4.6 Deep learning experiments

This research conducted four experiments to evaluate how different feature sets influence floodwater depth prediction. In each case, four image segmentation networks including FCN, UNet, UNet++, Attention-UNet were trained with consistent parameters (batch size = 8, epochs = 100 with early stopping, initial learning rate = 0.001 with decay, patch size = 256). Models were optimized with RMSE loss, and performance was reported using RMSE. five study sites, Lumberton, Goldsboro-1/2, Kinston-2 and Princeville from Hurricane Matthew, were selected for the following experiments;

• **Experiment 1 – Extent + Elevation:** Baseline setup combining inundation extent with ground elevation.
• **Experiment 2 – Experiment 1 + Man-made features:** Adds impervious surface and vegetation.
• **Experiment 3 – Experiment 1 + Terrain features:** Adds curvature, slope, and topographic wetness index.
• **Experiment 4 – All features:** Combines terrain and man-made features with extent and elevation.

Across experiments, UNet consistently outperformed the other models, with the largest performance gain observed when terrain features were included (Experiment 3). The best overall performance was achieved by UNet with all features (Experiment 4) (Table 7).

**Table 7:** RMSE test metrics for models across various experiments.

Model	Experiment 1 (m)	Experiment 2 (m)	Experiment 3 (m)	Experiment 4 (m)
FCN	0.42	0.42	0.35	0.36
U-Net	0.41	0.40	0.34	0.33
U-Net++	0.42	0.42	0.36	0.36
Attention U-Net	0.42	0.42	0.35	0.35

## Limitations

This dataset provides high resolution static (hydrostatic) inundation information suitable for a wide range of modeling, benchmarking and analysis particularly using data driven and deep learning approaches. However, the dataset is not designed for dynamic flow regimes (e.g., rapidly varying currents, breach waves, backwater transients, or critical channel flows). While dataset has been carefully processed and quality-controlled to ensure reliability, the unavoidable uncertainties such as sensor noise, DTM vertical bias, geo-registration drift, can result in commission/omission in flood-extent and water depth estimates. In addition, temporal mismatch among layers (e.g., imagery vs. later HWMs products) may yield inconsistencies; during recession, apparent pixel-wise depth errors should be treated as upper bounds. Finally, Flood2Depth was developed on selected sites with a natural class distribution (non-flooded > flooded), users should consider it when model training and evaluation for developing robust, generalizable approaches.

## Ethics Statement

The authors affirm that they followed the ethical requirements for publication in Data in Brief and that this work does not involve human subjects, animal experiments, or the use of data from social media platforms.

## CRediT Author Statement

**Jeffrey Blay:** Conceptualization, Data curation, Methodology, Validation, Writing-original draft, Writing-review & editing. **Yared Gebregziabher:** Methodology, Validation, Writing-original draft, Writing-review & editing. **Manoj K Jha:** Methodology, Writing-review & editing, Supervision. **Leila Hashemi-Beni:** Conceptualization, Methodology, Writing-review & editing, Resources, Supervision.

## Data Availability

ZenodoInundation2Depth Dataset (Original data)
